# Ethical and Legal Aspects of Organ Donation and Transplantation

**DOI:** 10.3389/ti.2024.13011

**Published:** 2024-04-09

**Authors:** Frederike Ambagtsheer, Coby Annema, John Forsythe, Nichon Jansen, David Paredes-Zapata

**Affiliations:** ^1^ Department of Internal Medicine, Nephrology and Kidney Transplantation, Erasmus MC Transplant Institute, University Medical Center Rotterdam, Rotterdam, Netherlands; ^2^ Department of Health Sciences, Section of Nursing Science, University of Groningen, University Medical Center Groningen, Groningen, Netherlands; ^3^ Department of Health and Social Services, Implementation Steering Group for Organ Utilisation, London, United Kingdom; ^4^ Department of Policy, Dutch Transplant Foundation, Leiden, Netherlands; ^5^ Donation and Transplant Coordination Section, Hospital Clínic, Barcelona, Spain; ^6^ Surgical Department, University of Barcelona, Barcelona, Spain; ^7^ Donation and Transplantation Institute Foundation, Barcelona, Spain

**Keywords:** transplant ethics, law, culture, psychology, bio-artificial organs

In organ transplantation, increasing emphasis is given to ethical and legal aspects. The persisting global organ shortage, in combination with fast-moving medical, technological, geopolitical and socio-economic changes, has given rise to an array of ethical and legal challenges for professionals working in the field of organ transplantation [1, 2]. This Special Issue provides a contemporary overview of ethical and legal considerations, both in deceased–and living organ donation and transplantation, reported by transplant professionals from across the globe. The publications do not only provide descriptions of current practices and considerations, but also offer inspiration and insight into how ethical, legal and cultural challenges can be overcome to further improve organ donation and transplantation rates around the world.

The first section of this issue focuses on ethical and legal challenges in deceased organ donation and transplantation, ranging from survey studies on opt-in -and opt-out systems Mihály et al. and radiological screening methods Chotkan et al. to difficulties in comparing deceased organ donation rates, even between countries that have similar cultures and organ donation systems Milross et.al. Many countries have changed their laws from an opt-in to a presumed consent system, among which the United Kingdom and Netherlands. Jansen et al. reflect on the experiences in these countries during these major changes, thereby offering valuable knowledge and guidance for professionals and policymakers who are considering changing national organ procurement laws. Rooted within discussions of organ procurement systems also lie cultural and religious considerations, which are highlighted in Atreya et al. contribution from Nepal. The authors offer solutions to how, among others, donation after brain death can be boosted in a country that faces considerable religious and cultural opposition to this form of donation.

The next publication focuses on directed donation after euthanasia Van Dijk et al. It is currently not possible to opt for directed donation following euthanasia. With more patients requesting deceased donation after euthanasia, Van Dijk et al. ask under which ethical considerations directed donation after euthanasia is ethically permissible. The authors offer a set of criteria under which it would be appropriate to proceed with directed donation following euthanasia. Another topic of debate is the question whether adults with impaired decision-making capacity should be allowed to be transplanted. In their literature review, Thom et al., on behalf of the ELPAT Working Group on Ethical and Legal Issues, describe how these adults face inequitable access to transplantation. They offer ethical and legal arguments, followed by recommendations, in support for allowing people with impaired decision-making capacity to be transplanted.

The second part of the Special Issue covers ethical and legal considerations in living organ donation and transplantation. It kicks off with a systematic review of reasons for and against anonymity in kidney paired donation by Marcus et al. This is followed by a unique interview study by Pronk et al. amongst unspecified donors in Netherlands who look back on their donation experiences. Next, Courtney et al. address, in their ELPAT paper, the issue that older patients are significantly less likely to receive a living donor transplant. The authors highlight the advantages of living donor transplantation in older patients, as well as systemic barriers, ethical, legal and social issues to explain the low representation of older individuals in living donor transplantation. Shifting from older donors to younger donors, Choi et al. evaluated the knowledge of and attitudes toward liver and kidney transplantation from minor donors in South Korea. They further assessed if receiving structured information on the outcomes of living organ transplantations and donations may change attitudes towards liver and kidney transplantation from minors. Several states in the United States, Canada, Belgium, Luxembourg, Norway, Sweden, the United Kingdom, Indonesia and South Korea allow donations of minors under exceptional circumstances Choi et al. Mamode et al. article explores issues raised by cases involving motivated living kidney donors whose willingness to take risk differs from that of the healthcare team. The authors explore the issues raised by these cases and consider the principles which might help to guide decision-making.

Following the rise of transplant tourism from Japan to China and the Declaration of Istanbul’s promulgation that transplant professionals have a duty to help prevent organ trade, several hospitals in Japan have announced that they will not provide follow-up care to patients suspected of participating in organ trafficking. Takimoto examines whether the refusal of follow-up care for transplant tourists is ethically acceptable, using two prevailing rationales—deterrent effect and conscientious objection. Although there have been calls for removing disincentives and allowing incentives for deceased–and living organ donation, there is limited information about their social acceptability. Ambagtsheer et al. present the results of a systematic literature review on public opinions towards removal of financial disincentives and the introduction of incentives for deceased and living organ donation in Europe. Next, they describe the results of a randomized survey experiment conducted on this issue in the United States. They propose this experiment’s framework as a blueprint for European research on this topic.

The final section of this Special Issue presents a cutting-edge topic in organ transplantation, namely the development of bio-artificial organs. To address the lack of ethical guidance for the safe and responsible design and conduct of early-phase clinical trials of bio-artificial organs, De Jongh et al. conducted a systematic review to examine the literature on early-phase clinical trials in these adjacent fields. They also present a thematic analysis of relevant ethical points to consider for early-phase clinical trials of transplantable bio-artificial organs. In this issue’s final study, Aguilera et al. present the results of an international survey among liver transplant providers regarding disparity and female leadership. The survey suggests that liver transplant providers may experience discrimination based on gender or race, lack of mentorship or support for discriminatory actions and very low rates of female representation in living transplant leadership positions, the lowest being in liver transplant surgery. The authors further identify higher rates of overall discrimination, discrimination in job promotion as well as compensation differences reported by female living transplant providers compared to male respondents. Several calls for action are proposed.

It is our hope that the combination of new empirical insights and ethical and legal guidelines presented in this issue offer a valuable framework for transplant professionals globally, be it to improve quality of patient care, to reduce inequity of access to transplantation or to reduce organ scarcity.
